# Middle Meningeal Artery Embolization Minimizes Burdensome Recurrence Rates After Newly Diagnosed Chronic Subdural Hematoma Evacuation (MEMBRANE): study protocol for a randomized controlled trial

**DOI:** 10.1186/s13063-022-06506-3

**Published:** 2022-08-22

**Authors:** Alexander Hoenning, Johannes Lemcke, Sergej Rot, Dirk Stengel, Berthold Hoppe, Kristina Zappel, Patrick Schuss, Sven Mutze, Leonie Goelz

**Affiliations:** 1 Center for Clinical Research, BG Klinikum Unfallkrankenhaus Berlin, Warener Str. 7, 12683 Berlin, Germany; 2 Department of Neurosurgery, BG Klinikum Unfallkrankenhaus Berlin, Warener Str. 7, 12683 Berlin, Germany; 3 BG Kliniken – Klinikverbund der Gesetzlichen Unfallversicherung gGmbH, Leipziger Pl. 1, 10117 Berlin, Germany; 4 Institute of Laboratory Medicine, BG Klinikum Unfallkrankenhaus Berlin, Warener Str. 7, 12683 Berlin, Germany; 5 Department of Radiology and Neuroradiology, BG Klinikum Unfallkrankenhaus Berlin, Warener Str. 7, 12683 Berlin, Germany; 6 Institute for Diagnostic Radiology and Neuroradiology, University Medicine Greifswald, Ferdinand-Sauerbruch-Straße, 17475 Greifswald, Germany

**Keywords:** Mild traumatic brain injury, Chronic subdural hematoma, Embolization, Surgery, Endovascular, Randomized controlled trial

## Abstract

**Background:**

Chronic subdural hematoma (cSDH) is the most common complication of mild traumatic brain injury demanding neurosurgery in high-income countries. If undetected and untreated, cSDH may increase intracranial pressure and cause neurological deficiencies. The first-line intervention of choice is burr hole trepanation and hematoma evacuation. However, any third patient may experience rebleeding, demanding craniotomy with excess morbidity. Adjunct endovascular embolization of the frontal and parietal branches of the middle meningeal artery (MMA) is a promising approach to avoid relapse and revision but was hitherto not studied in a randomized trial.

**Methods:**

MEMBRANE is an investigator-initiated, single-center, randomized controlled trial. Male, female, and diverse patients older than 18 years scheduled for surgical evacuation of a first cSDH will be assigned in a 1:1 fashion by block randomization to the intervention (surgery plus endovascular MMA embolization) or the control group (surgery alone). The primary trial endpoint is cSDH recurrence within 3 months of follow-up after surgery. Secondary endpoints comprise neurological deficits assessed by the modified Rankin Scale (mRS) and recurrence- or intervention-associated complications during 3 months of follow-up. Assuming a risk difference of 20% of rebleeding and surgical revision, a power of 80%, and a drop-out rate of 10%, 154 patients will be enrolled onto this trial, employing an adaptive O’Brien-Fleming approach with a planned interim analysis halfway.

**Discussion:**

The MEMBRANE trial will provide first clinical experimental evidence on the effectiveness of endovascular embolization of the MMA as an adjunct to surgery to reduce the risk of recurrence after the evacuation of cSDH.

**Trial registration:**

German Clinical Trials Registry (Deutsches Register Klinischer Studien [DRKS]) DRKS00020465. Registered on 18 Nov 2021. ClinicalTrials.gov NCT05327933. Registered on 13 Apr 2022.

## Administrative information

Note: the numbers in curly brackets in this protocol refer to SPIRIT checklist item numbers. The order of the items has been modified to group similar items (see http://www.equator-network.org/reporting-guidelines/spirit-2013-statement-defining-standard-protocol-items-for-clinical-trials/).

**Table:** 

Title {1}	Middle Meningeal Artery Embolization Minimizes Burdensome Recurrence Rates After Newly Diagnosed Chronic Subdural Hematoma Evacuation (MEMBRANE): study protocol for a randomized controlled trial
Trial registration {2a and 2b}.	Trial identifier 1: DRKS00020465 Registry name: German Clinical Trials Registry (Deutsches Register Klinischer Studien [DRKS]) Trial identifier 2: NCT05327933 Registry name: ClinicalTrials.gov
Protocol version {3}	Date: 19 Apr 2022, Version 5.0
Funding {4}	The trial is funded by the German Social Accident Insurance (Deutsche Gesetzliche Unfallversicherung e.V. [DGUV])
Author details {5a}	Hoenning Alexander^1^, Lemcke Johannes^2^, Rot Sergej^2^, Stengel Dirk^3^, Hoppe Berthold^4^, Zappel Kristina^1^, Schuss Patrick^2^, Mutze Sven^5,6^, Goelz Leonie^5,6^ Affiliations: ^1^Center for Clinical Research, BG Klinikum Unfallkrankenhaus Berlin, Warener Str. 7, 12683 Berlin, Germany ^2^Department of Neurosurgery, BG Klinikum Unfallkrankenhaus Berlin, Warener Str. 7, 12683 Berlin, Germany ^3^BG Kliniken – Klinikverbund der Gesetzlichen Unfallversicherung gGmbH, Leipziger Pl. 1, 10117 Berlin ^4^Institute of Laboratory Medicine, BG Klinikum Unfallkrankenhaus Berlin, Warener Str. 7, 12683 Berlin, Germany ^5^Department of Radiology and Neuroradiology, BG Klinikum Unfallkrankenhaus Berlin, Warener Str. 7, 12683 Berlin, Germany ^6^Institute for Diagnostic Radiology and Neuroradiology, University Medicine Greifswald, Ferdinand-Sauerbruch-Straße, 17475 Greifswald, Germany
Name and contact information for the trial sponsor {5b}	Investigator initiated trial, principal investigator: Johannes Lemcke (JL), Department of Neurosurgery, BG Klinikum Unfallkrankenhaus Berlin, Warener Str. 7, 12683 Berlin, Germany, johannes.lemcke@ukb.de, Phone +49 30 5681 3701
Role of sponsor {5c}	The trial sponsor, represented by JL, is responsible for all aspects of conducting the trial including its design, data collection, management, analysis, interpretation of data, reporting results and the decision to submit the report for publication. Data safety is overseen by an independent Data Safety Monitoring Board (DSMB) consisting of a neuroradiologist, a neurosurgeon, and a clinical epidemiologist not involved in the trial.

## Introduction

### Background and rationale {6a}

Chronic subdural hematoma (cSDH) is the most frequent complication of mild traumatic brain injury requiring neurosurgical care, and the most common type of traumatic intracranial hemorrhage in high-income countries alike [1]. The reported annual incidence of cSDH ranges from 1.7 to 20.6 per 100,000 [2], with a significant increase noted during the past years. The latter is attributed to a more liberal use of computed tomography (CT) and magnetic resonance imaging (MRI), as well as to demographic changes [3].

Pathogenesis of cSDH comprises fibrinolysis and liquefication of the initial blood clot, secondary local inflammatory response, and subsequent formation of space-occupying subdural neo-membranes up to ten days after injury [4, 5]. Apart from older age, known risk factors of cSDH are male gender, therapeutic anticoagulation and bleeding disorders, alcohol abuse, diabetes mellitus, and arterial hypertension [6–8]. Specifically, subnormal factor XIII activity due to polymorphism in coding genes F13A1 rs2815822 [9] and F13B rs12134960 [10] is associated with an enhanced risk of postoperative intracranial bleeding [11].

Non-operative management with serial cranial CT monitoring may be suitable for small, asymptomatic hematomas, while symptomatic cSDH almost always needs neurosurgical intervention [12]. Burr hole trepanation is regarded as the current standard of care, with acceptable morbidity and mortality [13–16]. Still, recurrent cSDH occurs in up to one-third of cases after surgery [17, 18].

A meta-analysis of 250 studies enrolling 34829 patients showed substantial heterogeneity due to varying definitions of recurrence [13]. In a retrospective analysis of patients younger than 45 years with cSDH, the incidence of recurrent bleeding was estimated at 7/29 (24%) [19].

Several options were proposed to lower the risk of recurrence and reoperation (i.e., Ommaya reservoirs placed in the subdural space, endoscopically-assisted surgery, systemic carbazochrome, or glucocorticoids), none of which turned out to be markedly effective.

Embolization of the middle meningeal artery (MMA) by polyvinyl alcohol (PVA) particles, liquid embolic agents such as Onyx® (Medtronic, Dublin, Ireland), and/or coiling is a promising interventional adjunct to surgery for cSDH [17, 20, 21], which may also prevent micro-bleeding from cSDH membranes [17]. It has been established as a safe procedure [16, 17, 20, 22–27] in clinical practice for various other indications (e.g., dural arteriovenous fistulas, meningiomas) [28, 29]. Although single cases of cranial nerve palsy and blindness were reported [30, 31], the risk of adverse events may be minimized by excluding anastomoses between intra- and extracranial vessels [32].

Ban et al. conducted an observational study of primary endovascular management of patients with cSDH compared to a historical cohort undergoing the standard of care [20]. Recurrence risks were 1/72 (1.4%) and 129/469 (27.5%), respectively, for an adjusted odds ratio [OR] of 0.056 (95% CI 0.011 to 0.286). Patient selection was conditional on space-occupation by cSDH, with a higher likelihood of surgery with larger blood collections.

The Middle Meningeal Artery Embolization Minimizes Burdensome Recurrence Rates After Newly Diagnosed Chronic Subdural Hematoma Evacuation (MEMBRANE) trial aims at investigating the effectiveness and safety of supportive MMA embolization after surgical evacuation of cSDH.

### Objectives {7}

#### Primary objective

The primary objective of this study is to investigate whether surgical hematoma evacuation plus endovascular embolization of the MMA lowers the risk of cSDH recurrence compared to surgery alone.

#### Secondary objectives

Secondary objectives of this study include the assessment of

- Neurological deficits,
- Recurrence-associated complications, and
- Complications associated with endovascular embolization.

#### Tertiary objectives

Tertiary objectives of the study are to investigate

- The relationship between factor XIII deficiency and risk of recurrence and
- The association between the genetic variants F13A1 rs2815822 and F13B rs12134960 with factor XIII activity.

### Trial design {8}

**Fig. 1:**
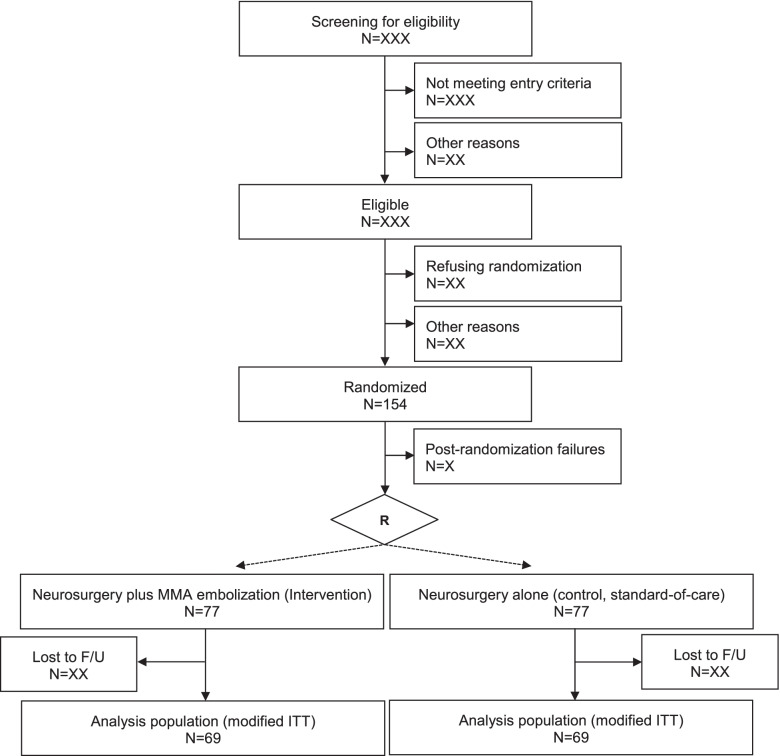
Study flowchart

MEMBRANE is an investigator-initiated, single-center, randomized controlled trial testing whether neurosurgical hematoma evacuation plus endovascular embolization of the MMA (intervention group) is superior to surgery alone (control group) in reducing the recurrence rate of cSDH within a follow-up period of 3 months. Eligible patients will be allocated in a 1:1 ratio to either the intervention or control group (see study flowchart in Fig. 1).

## Methods: participants, interventions, and outcomes

### Study setting {9}

The MEMBRANE trial will be conducted at the BG Klinikum Unfallkrankenhaus Berlin (ukb) in Berlin, Germany. The ukb is a tertiary care, academic teaching hospital, and certified supra-regional trauma center with more than 730 beds, 17 operating theatres, a dual-use helicopter, and one of the busiest interdisciplinary stroke units in the capital of Germany. Given its mobile stroke unit and wide-ranging tele-neurology service, the ukb emerged as a premier national referral institution for acute neurological and neurosurgical cases [33, 34].

All clinical investigators are board-certified and have renowned expertise in neurosurgery, neuroradiology, and endovascular interventions. Structural and process quality in terms of state-of-the-art radiological imaging, surgery, critical care, and management of complications are provided, subject to federal quality assurance and maintenance measures. Research is facilitated by a distinct methodological center, guaranteeing compliance with and adherence to ICH-GCP, data safety measures (i.e., EU-GDPR), and all relevant ethical and reporting standards.

### Eligibility criteria {10}

#### Inclusion criteria

1. Patients scheduled for surgery by means of one or more burr hole trepanations during the first manifestation of a cSDH (unihemispherical or bihemispherical), as detected by CT or MRI
2. Age ≥18 years
3. Written informed consent of the patient to participate in the trial
4. Likely compliance of the participant in attending follow-up examination

#### Exclusion criteria

1. Conservatively treated cSDH
2. Age <18 years
3. Radiological evidence of an acute or subacute subdural hematoma, subarachnoid hemorrhage, intracerebral hematoma, or epidural hematoma
4. Primary craniotomy, craniectomy, or bilateral burr hole trepanation
5. Angiography cannot be performed within 72 h after surgery
6. Supervisory relationship
7. Pregnancy
8. Lack of informed consent
9. Likely lack of compliance
10. Homozygous factor XIII activity <10%

### Who will take informed consent? {26a}

The investigator or her/his representative (at least a board-certified physician) of the Department of Neurosurgery or Department of Radiology and Neuroradiology at the ukb will approach eligible patients, hand out an informed consent form, and explain (in plain language) the rationale, design, risks, and potential benefits of the study. Patients will be given ample of time to ask questions, and are requested to provide written informed consent within 24 h.

### Additional consent provisions for collection and use of participant data and biological specimens {26b}

Patients willing to participate in the sub-study on factor XIII activity of this trial must provide extra consent for genetic analyses in accordance with the German Act of Genome Diagnostics (“Gendiagnostikgesetz”, https://www.bundesgesundheitsministerium.de/service/begriffe-von-a-z/g/gendiagnostikgesetz.html).

Relevant patient information and consent forms were approved by the IRB (Charité University Medicine Berlin, Germany, EA1/119/19) in October 2019.

## Interventions

### Explanation for the choice of comparators {6b}

Patients assigned to the control group will be managed according to the current standard of care, i.e., neurosurgical evacuation of cSDH by burr hole trepanation and/or any surgical technique without specific added treatment.

### Intervention description {11a}

**Fig. 2:**
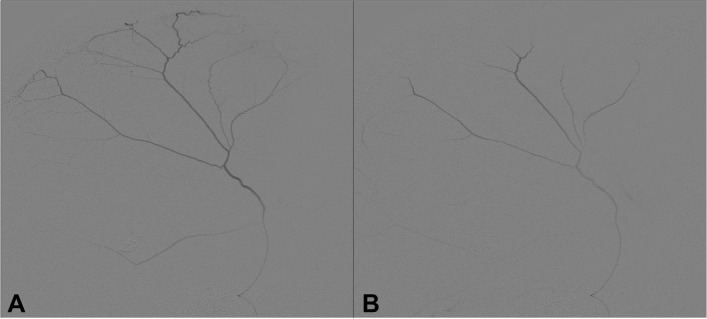
Lateral angiograms of the MMA before embolization (**A**) and after injection of 0.5 ml PVA particles (100–300μm) obstructing the peripheral branches of the MMA (**B**)

Patients assigned to the experimental arm will undergo neurosurgical evacuation of cSDH by burr hole trepanation and/or any surgical technique plus endovascular embolization of the MMA (see Fig. 2).

The embolization procedure can briefly be described as follows: A micro-catheter is inserted via a transfemoral approach into the branches of the MMA in a minimally invasive manner, and the periphery is occluded using PVA particles to prevent future bleeding. For this purpose, the micro-catheter is positioned as distally as possible, which prevents dislocation of particles into nutritive branches [29, 35]. If the desired catheter position cannot be achieved due to anatomical conditions, the MMA can optionally be closed more proximally using Onyx® or micro-electric coils. Embolization of the MMA by PVA particles with sizes between 40 and 300 μm is preferred over embolization by coils and Onyx®, since the capillary network of the dura is entirely blocked when using particles [35]. After using coils, a faster reperfusion via collaterals might be possible.

Target vessels are identified by digital subtraction angiography (DSA) in patients assigned to the intervention group, meaning increased exposure to radiation. Based on embolization of MMA branches in meningioma patients, a dose area product (DAP) of about 6000 to 16,000 centi-gray centimeter squared (cGy per cm^2^) is expected depending on the extent of embolization and individual anatomy. Thus, it is very much unlikely the DAP associated with the experimental adjunct exceeds the reference value for neuroradiological interventions of 20,000 to 30,000 cGy per cm^2^ as defined by the Federal Office for Radiation Protection of Germany (“Bundesamt für Strahlenschutz”, BfS). The trial and imaging protocol was approved by the BfS on March 25, 2022, under notification reference ZD 3-22464/2022-015-A.

### Criteria for discontinuing or modifying allocated interventions {11b}

Patients may withdraw their consent at any time without providing a reason and thus terminate their participation in the study prematurely. Withdrawal from the study and reasons, if known, will be documented. Criteria for premature drop-out include:

- Subsequent occurrence of an exclusion criterion
- Loss of contact
- Death
- Declaration of withdrawn consent

Moreover, the principal investigator is entitled to terminate the study prematurely if:

- Patient recruitment remains inadequate despite multiple measures to improve enrolment
- Serious problems with the quality of collected data cannot be resolved
- Unforeseeable circumstances at the trial center prevent trial continuation
- Unacceptable risks arise (after a risk-benefit assessment by the Data Safety Monitoring Board)
- New scientific findings in favor of one or the treatment violate the equipoise principle

### Strategies to improve adherence to interventions {11c}

There is no possibility to influence patients’ adherence to the intervention. Embolization of the MMA constitutes the only study intervention, which is solely performed by the clinical investigators.

### Relevant concomitant care permitted or prohibited during the trial {11d}

Except for the study intervention, patients in both groups are treated according to the currently established standard of care at the trial center (ukb). Any concomitant care as part of routine clinical practice is permitted.

### Provisions for post-trial care {30}

A proband cover for all patients participating in the study is contracted to compensate for trial-associated harm occurring within 5 years of trial participants’ final study visit.

### Outcomes {12}

#### Primary outcome

The primary outcome is cSDH recurrence up to 3 months after surgery. Recurrence is defined by one and/or both of the following criteria:

- Recurrent cSDH with at least the same volume (±10%) compared to baseline findings
- Recurrent cSDH which requires surgery

Recurrence of cSDH is operationalized as a binary outcome (i.e., recurrence or no recurrence) during a follow-up period of 3 months.

#### Secondary outcomes

The following secondary outcomes will be assessed up to 3 months after surgery:

- Neurological deficits evaluated by the modified Rankin scale (mRS)
- Quantity and characteristics of recurrence-associated complications
- Quantity and characteristics of complications associated with endovascular embolization

The mRS [36] measures the degree of disability or dependence in the daily activities of people who have suffered a stroke or other causes of neurological disability. Scale values and interpretations are:

- 0 = No symptoms.
- 1 = No significant disability. Able to carry out all usual activities, despite some symptoms.
- 2 = Slight disability. Able to look after own affairs without assistance, but unable to carry out all previous activities.
- 3 = Moderate disability. Requires some help, but able to walk unassisted.
- 4 = Moderately severe disability. Unable to attend to own bodily needs without assistance, and unable to walk unassisted.
- 5 = Severe disability. Requires constant nursing care and attention, bedridden, incontinent.
- 6 = Dead

#### Tertiary outcomes

The following tertiary outcomes are only addressed in a sub-sample of participants consenting to genetic analyses:

- Relationship between factor XIII deficiency (i.e., activity after cryopreservation <70%) and risk of cSDH recurrence
- Predisposition of genetic variants F13A1 rs2815822 and F13B rs12134960 for factor XIII deficiency

### Participant timeline {13}

Participants in the intervention and control groups will undergo five and four scheduled follow-up visits, respectively (Table 1):

*−t*_1_ Screening and informed consent
- *t*_0_ Randomization
- *t*_1_ Baseline
- *t*_2_ Embolization (intervention group only, within 72 h after surgery)
- *t*_3_ Follow-up 1 month after baseline (± 1 week)
- *t*_4_ Follow-up 3 months after baseline (± 1 week)

**Table 1:** Schedule of enrollment, interventions, and assessments

	Study period					
	Enrollment	Allocation	Post-Allocation			Close-Out
Time point	*−t*_*1*_	*t*_*0*_	*t*_*1*_	*t*_*2*_	*t*_*3*_	*t*_*4*_
**Enrollment**						
Eligibility screen	X					
Informed consent	X					
Randomization		X				
**Intervention**						
Embolization				X		
**Assessments**						
Demographics, medical history, laboratory data			X			
Neurological tests			X		X	X
Computed tomography			X		X	X
Complications				X	X	X

### Sample size {14}

Because of the potential of notable morbidity, recurrent cSDH is considered both a clinically relevant and methodologically reliable, objective primary trial endpoint.

In one of the largest studies to date, Ban et al. compared a prospective series of 72 patients with cSDH undergoing MMA embolization with a historic standard-of-care group of 469 subjects. The overall risk difference (irrespective of hematoma evacuation) was estimated at 26% (95% CI 21 to 31%) in favor of endovascular management. There is much variability in reported cSDH recurrence risks with either treatment option, and unclarity about the realistic benefit of combined surgical and interventional therapy.

In view of the results of published studies (in particular Ban et al.), we conservatively presume recurrence rates of 30% in the control and 10% in the experimental arm during an observation period of 3 months.

Since our assumptions are subject to uncertainty, we will employ an adaptive design according to O’Brien-Fleming with one planned interim analysis to decide about the further trial progress, and to modify the target sample size if necessary and/or reasonable. For a power of 80% and a total alpha of 5%, data from 138 patients (i.e., 69 per group) are needed to detect a risk difference of 20% by a *z*-test for independent samples. Assuming a drop-out and lost-to-follow-up rate of 10%, we plan to enroll 154 patients (i.e., 77 per treatment arm) unless the interim look prompts any adjustment.

### Recruitment {15}

During a kick-off meeting, clinical investigators and trial supporting personnel will be trained in communicating with potential study participants and their relatives, documentation including screening logs, and other standard operating procedures established for trial purposes.

In agreement with the funding body, recruitment efficiency will be evaluated 9, 15, and 21 months after randomization of the first patient, using the observed to expected ratio and curve.

In case of insufficient enrollment (i.e., <25% of the number of patients included at a certain time point), investigators will take extra measures to improve recruitment (e.g., increasing awareness among all clinicians, nurses, therapists, expansion of the recruiting team, modification of eligibility criteria, etc.). While it is currently not intended to roll out this trial to other centers, we will negotiate with the funding body to include other institutions if trial aims cannot be achieved otherwise.

## Assignment of interventions: allocation

### Sequence generation {16a}

Patients will randomly be assigned to trial arms using a web platform and an extended stratified block algorithm. The algorithm guarantees even distribution of key baseline characteristics across the intervention and the control group.

### Concealment mechanism {16b}

Participants are randomized using secuTrial® (interActive Systems GmbH, Berlin, Germany), a web-based, GCP-compliant electronic data capture (EDC) system for collecting patient data in clinical trial, observational studies, and registries. It maintains allocation concealment as it does not release the randomization code until screening has been completed and the patient was cleared to be recruited onto the trial.

### Implementation {16c}

Extended stratified block algorithms generate an unpredictable allocation sequence. Random assignment by secuTrial® cannot be influenced by clinical investigators.

## Assignment of interventions: blinding

### Who will be blinded {17a}

Due to the surgical and interventional nature of trial modalities, blinding of both the immediately performing clinical investigators (i.e., neurosurgeons and interventional radiologists) and patients is almost impossible. While a sham procedure (i.e., placement of a transfemoral catheter and even infusion of a saline placebo) in the control group may be implemented, this is considered unethical as it exposes patients to additional harm without any possible benefit, given the primary endpoint.

Radiological experts evaluating baseline and follow-up CT scans after 1 and 3 months will be blinded to the randomly assigned treatment, assuring this is an observer-blinded trial.

### Procedure for unblinding if needed {17b}

If radiologists note any irregularity or cumulation of cSDH recurrences among trial participants during follow-up, this may lead to unblinding of treatment assignment after consultation of the DSMB.

## Data collection and management

### Plans for assessment and collection of outcomes {18a}

Data will be entered by clinical investigators and supporting trial personnel on electronic case report forms (eCRFs) created by secuTrial®.

Neurological examination comprises the modified Rankin scale for neurologic disability [36], the muscle function according to Janda [37], the presence of word finding disorders, hemiparesis, and anisocoria, as well as vigilance. Standard laboratory parameters will be taken from peripheral blood at baseline and during follow-up. The size and extent of cSDH will be measured on non-contrast CT scans.

The following parameters of recurrence-associated or intervention-associated complications will be documented for study purposes:

Recurrence-associated:

- Cerebral edema
- Cerebral infarction

Intervention-associated:

- Dissection of vessels by the guide wire
- Dislocation of particles or coils
- Ischemia caused by internal-external vascular connections
- Retinal blindness
- Epidural hematoma after MMA perforation
- Inflammation or abscess at the puncture site
- Vascular fistula at the puncture site
- Vascular bulging at the puncture site
- Allergic reaction to the contrast agent
- Acute renal failure

Complications not covered by predefined categories may be entered as free text in the eCRF.

### Plans to promote participant retention and complete follow-up {18b}

Neurosurgical hematoma evacuation and endovascular MMA closure represent standard modalities both at the trial site and in the scientific community- the novelty of MEMBRANE is random assignment to surgery plus endovascular treatment versus surgery alone. Patients will deliberately be informed that trial participation guarantees no personal benefit whatsoever- however, it will also be explained there is scientific evidence that patients treated in a clinical trial environment may show superior outcomes compared to those managed under routine practice conditions.

Investigators, study nurses and other staff will take care of retaining participants until the scheduled last follow-up visits.

Patients who chose to discontinue participation, those who do not respond to multiple attempts of contact or cannot be reached by phone or mail will be considered losses to follow-up. Data will be recorded, stored, and used for final analysis unless participants actively demand data deletion. Those appeals will be handled by local data security officers in accordance with EU-GDPR.

### Data management {19}

Electronic case report forms (eCRF) will be computed in secuTrial® (interActive Systems Berlin, Germany). The system fully complies with ICH-GCP and FDA 21 CFR Part 1, is hosted by the Center for Clinical Research at the trial institution and was successfully used for data storage, exchange, and processing in multiple clinical trials and observational studies. Dedicated data managers are responsible for programming eCRFs and quality maintenance.

Investigators and trial staff will be introduced to the platform and trained in data entry during the initial kick-off meeting prior to recruitment of the first patient.

Only authorized clinical investigators and trial personnel will be granted access to the study database by a personal ID. Depending on their role within the study, users will be assigned tailored authorizations to the respective forms.

All study data are stored and processed in a pseudonymized fashion. For data transmission between the ward and peripheral computers and the study database, a 128-bit SSL connection is used for data encryption to avoid manipulation. Each time data is entered into secuTrial®, an audit trail is created which guarantees complete versioning and traceability of changes. Customized field properties, rules, and checks in secuTrial®, which indicate the input of implausible values, inconsistent information, or the lack of mandatory information, help to optimize data quality. Once data entry is complete, the database will be closed and data exported for statistical analysis.

### Confidentiality {27}

secuTrial® uses a pseudonymization concept that completely separates personal from identifying and medical data. When adding a new patient to the database, identifying data are entered on a form which is only printed but not saved on the server. On this form, the so-called participant identification list, a pseudonym consisting of a combination of six alphanumeric characters is automatically created for each patient. The form is kept in a locked space to which only the principal investigator has access and may be used to unblind personal data if necessary.

### Plans for collection, laboratory evaluation, and storage of biological specimens for genetic or molecular analysis in this trial/future use {33}

Blood drawn from patients at baseline will routinely be analyzed for platelet count, activated partial thromboplastin time (aPTT), and fibrinogen concentration. For study purposes, we will analyze factor XIII concentration by means of STA-R (Stago, Düsseldorf, Germany) with HEXAMATE Factor XIII (Roche Diagnostics, Basel, Switzerland), and the presence of genetic variants F13A1 rs2815822, F13B rs12134960, F13A1 rs2815822 (C > A) and F13B rs12134960 (C > G). To determine factor XIII activity, blood samples will be cryopreserved for later analysis.

## Statistical methods

### Statistical methods for primary and secondary outcomes {20a}

The primary outcome, i.e., reduction in cSDH recurrence through the experimental, surgical plus endovascular treatment, compared to surgery alone, will be analyzed using a chi-square fourfold test for independent samples. The two-sided significance level *α* is set at 5% and the power at 80%. Superiority of the experimental intervention over control is assumed, if the resulting *p*-value is below the nominal significance level of 0.00031 at the time of interim analysis (prompting early trial termination), or the nominal significance level of 0.0469 at the time of final analysis.

Secondary categorical outcomes will be investigated by the Cochran-Mantel-Haenszel (CMH), chi-square, or Fisher’s exact test (if one of the cell frequencies is <5). Recurrence-associated complications, complications associated with endovascular embolization, and (serious) adverse events will be expressed as absolute and relative frequencies.

Exploratory analyses will be performed for the distribution of genetic variants and their correlation (by Pearson’s *phi*-coefficient) with factor XIII deficiency (<70%) across the entire cohort without stratification for the type of intervention. The closer the *phi*-coefficient to 1 or -1, the stronger the association (or predisposition) between genetic variants and factor XIII deficiency. Advanced analyses will be performed to study the association between heterozygous, homozygous, and wild type variants, factor XIII activity, and cSDH recurrence.

### Interim analyses {21b}

The planned interim analysis according to the O’Brien-Fleming design will be conducted after the recruitment of half of the target sample. Given the predicted recruitment speed and rate will be reached on time, the interim analysis will be conducted 12 months after first-patient-in (plus 3 months of follow-up), corresponding to 77 included patients and 69 evaluable data sets. If a statistically significant advantage in the recurrence rate of the experimental group can be demonstrated at the time of the interim analysis with a *p*-value of the chi-squared test of <0.0031, the experimental therapy will be assumed to be superior to the standard of care, leading to premature termination of the study. If the effect size is lower than presumed, a larger sample size and enrolment of additional trial sites must be considered. Investigators will discuss this option together with the funding body and the DSMB. If the likely risk difference between treatments is much smaller than expected and the recalculated sample size exceeds numbers which cannot be achieved, the trial will be terminated for futility. The trial will also be terminated early if the surgery-only control group turns out to be superior to combined management in any case, or if major novel scientific evidence shows any substantial favor of one over the other approach.

### Methods for additional analyses (e.g., subgroup analyses) {20b}

Primary and secondary outcomes will be stratified for age in a categorial manner (i.e., ≤67 and >67 years, the common retirement age in Germany).

### Methods in analysis to handle protocol non-adherence and any statistical methods to handle missing data {20c}

The primary analysis will be performed on the modified intent-to-treat (mITT) set including all randomized patients and based on the treatment arm they were randomized to, regardless of the therapy they actually received. The modification of the ITT set implies that study outcomes must be fully documented at the follow-up visit at 3 months. The per-protocol (PP) set is used for sensitivity analyses, including participants who were treated in full accordance with the study protocol and actually received the randomly assigned treatment.

A missing data analysis will be conducted investigating the extent and type of missing values in trial endpoint variables. In case of a suspected non-random missing data mechanism (e.g., Missing At Random [MAR], Missing Not At Random [MNAR]), corresponding sensitivity analyses will be carried out along with a discussion of the results. If suitable, we will consider multiple imputation (MI) to fill empty cells in the dataset.

### Plans to give access to the full protocol, participant-level data and statistical code {31c}

The trial was registered prospectively on 18 Nov 2021 (http://www.drks.de/DRKS00020465) with the German Clinical Trials Registry (Deutsches Register Klinischer Studien [DRKS]) as a primary registry of the WHO International Clinical Trials Registry Platform (ICTRP; https://trialsearch.who.int/). In addition, the trial will appear in ClinicalTrials.gov with the identifier NCT05327933. Updates will be submitted if significant milestones have been reached.

## Oversight and monitoring

### Composition of the coordinating center and trial steering committee {5d}

Since this is a single-center trial not regulated by German laws on pharmaceuticals (“Arzneimittelgesetz”) or medicinal products (“Medizinproduktegesetz”), a steering committee is not deemed necessary. Regular meetings of the principal investigator, the two study coordinators from the Department of Neurosurgery and the Department of Radiology and Neuroradiology, a clinical investigator from the Institute of Laboratory Medicine, and a research associate from the Center for Clinical Research will be scheduled to ensure high process quality and compliance with the study protocol. Participating clinicians must take part in a training course as part of a kick-off event initiated by the principal investigator. Each participant must confirm in written form that she/he was properly introduced to trial-specific procedures.

A Data Safety Monitoring Board (DSMB) will be established for evaluating serious adverse events (SAE).

Study participants are treated at departments of maximum care and proven neurosurgical and neuroradiological expertise. Responsible interventional radiologists have many years of experience in the use of PVA particles, liquid embolic agents (Onyx®), and coils to occlude extracranial vessels. They possess level 2 certificates in modules E and F of the German Society for Interventional Radiology and Minimally Invasive Therapy (DeGIR)/German Society for Neuroradiology (DGNR).

The Institute for Laboratory Medicine at the trial institution is accredited to analyze genetic factor XIII variants, and qualified for genetic counseling in accordance with Section 7 (3) of the German Genetic Diagnostics Act (GenDG). All DeGIR/DGNR certificates as well as the qualifications for genetic counseling of the participating investigators are stored in the trial master file (TMF).

### Composition of the data monitoring committee, its role, and reporting structure {21a}

The DSMB will regularly receive blinded statistical reports and monitor serious adverse events (SAEs) throughout the trial, decide whether patient safety is compromised, demanding premature closure of the trial. The independent panel consists of two clinical experts and a clinical epidemiologist not employed by the ukb.

### Adverse event reporting and harms {22}

All SAEs reported by study participants or observed by an investigator within the study period must be documented in the eCRF. SAEs are any undesirable sign, symptom, or medical condition which

- Is fatal
- Is acutely life-threatening
- Requires or prolongs inpatient hospitalization
- Results in persistent or significant disability/incapacity
- Constitutes a congenital anomaly or birth defect (not applicable, pregnancy excludes from study participation)

The principal investigator will be informed immediately about the onset of a SAE and must decide whether the participant shall be excluded from or remain in the study. The likelihood of a causal relationship between the individual trial intervention and SAE will be investigated further, and patients will be observed until the SAE resolves or a stable condition is reached.

### Frequency and plans for auditing trial conduct {23}

Data management staff will stay in regular contact with the investigators about trial progress, data consistency, missing data, and time window violations. If necessary, data queries for missing data as well as clarifications of inconsistencies or discrepancies will be sent.

### Plans for communicating important protocol amendments to relevant parties (e.g., trial participants, ethical committees) {25}

Any change in the clinical investigation plan, experimental and control interventions, follow-up scheme, etc. must be cleared in written form and signed by all persons in charge, stating the reasons for changes. Subsequently, these changes will be considered part of the study protocol. If necessary, changes must also be approved by the IRB and/or individual participants. Changes must be filed as an amendment in the TMF.

### Dissemination plans {31a}

After database closure, a biometric report will be written by the trial statistician describing the main study results. Subsequently, a meeting among investigators and collaborators will be held to discuss findings prior to drafting of a scientific manuscript to be submitted for peer-review and publication in a major scientific journal. Also, we will attempt to present results at key international conferences of neurosurgical and radiological societies.

## Discussion

We herein describe the rationale, design, interventions, and methodological framework of a single-center, investigator-initiated, partially blinded, randomized controlled trial to test whether additional endovascular embolization of the MMA is superior to neurosurgical hematoma evacuation only in reducing the rate of recurrence in cSDH.

Current observational evidence suggests that postoperative occlusion of MMA branches may reduce the incidence of recurrent cSDH without major complications. Yet, a randomized controlled trial is urgently needed to evaluate the benefit-to-risk ratio of additional embolization of the MMA compared to the neurosurgical standard of care.

If combined surgical and interventional care turns out to be superior to surgery alone in this trial, this may prompt further confirmatory trials and, at best, may change clinical practice and guideline recommendations.

## Trial status

This manuscript is based on trial protocol version 5.0, dated 19 Apr 2022. At the time of submission, patient recruitment has not yet begun. Recruitment of patients is planned to start on 20 Apr 2022. Recruitment is estimated to be completed in the third quarter of 2024.

## Data Availability

Data will be made available from the corresponding author upon reasonable request.

## Abbreviations

AHQR: Agency for Healthcare Research and Quality
AMG: Medicinal Products Act
aPTT: Activated partial thromboplastin time
cGy per cm^2^: Centi-gray centimeter squared
CMH: Cochran-Mantel-Haenszel
cSDH: Chronic subdural hematoma
CT: Computed tomography
DAP: Dose area product
DeGIR: German Society for Interventional Radiology and Minimally Invasive Therapy
DGNR: German Society for Neuroradiology
DRKS: Deutsches Register Klinischer Studien
DGUV: German Legal Accident Insurance
DSA: Digital subtraction angiography
DSMB: Data Safety Monitoring Board
eCRF: Electronic case report form
EDC: Electronic data capture
GCP: Good Clinical Practice
ICTRP: International Clinical Trials Registry Platform
ITT: Intent-to-treat
μm: Micrometer
MAR: Missing At Random
MEMBRANE: Middle Meningeal Artery Embolization Minimizes Burdensome Recurrence Rates After Newly Diagnosed Chronic Subdural Hematoma Evacuation
MI: Multiple imputation
mITT: Modified intent-to-treat
MMA: Middle meningeal artery
MNAR: Missing Not At Random
MPG: Medical Devices Act
mRS: Modified Rankin scale
PP: Per-protocol
PVA: Polyvinyl alcohol
SAE: Serious adverse event
T: Time point
TMF: Trial master file
ukb: BG Klinikum Unfallkrankenhaus Berlin gGmbH
